# Characterization of Graft Copolymers Synthesized from *p*-Aminosalicylate Functionalized Monomeric Choline Ionic Liquid

**DOI:** 10.3390/pharmaceutics15112556

**Published:** 2023-10-30

**Authors:** Aleksy Mazur, Dorota Neugebauer

**Affiliations:** Department of Physical Chemistry and Technology of Polymers, Faculty of Chemistry, Silesian University of Technology, 44-100 Gliwice, Poland; aleksy.mazur@polsl.pl

**Keywords:** choline methacrylate, biofunctionalized monomeric ionic liquid, *p*-aminosalicylate, ionic graft copolymers, ionic conjugate, drug delivery system

## Abstract

An ionic liquid based on the monomeric choline, specifically [2-(methacryloyloxy)ethyl]-trimethylammonium chloride (TMAMA), underwent biofunctionalization through an ion exchange reaction with the model drug anion: *p*-aminosalicylate (PAS), a primary antibiotic for tuberculosis treatment. This modified biocompatible IL monomer (TMAMA/PAS) was subsequently copolymerized with methyl methacrylate (MMA) to directly synthesize the well-defined graft conjugates with regulated content of ionic fraction with PAS anions (up to 49%), acting as drug delivery systems. The length of the polymeric side chains was assessed by the monomer conversions, yielding a degree of polymerization ranging from 12 to 89. The density of side chains was controlled by “grafting from” using the multifunctional macroinitiators. In vitro drug release, triggered by the ion exchange between the pharmaceutical and phosphate anions in a PBS medium, occurred in the range of 71–100% (2.8–9.8 μg/mL). Owing to significant drug content and consistent release profiles, these particular graft copolymers, derived from biomodified IL monomers with ionically attached pharmaceutical PAS in the side chains, are recognized as potentially effective drug delivery vehicles.

## 1. Introduction

The delivery of biologically active substances via polymeric materials has become one of the most focused upon research trends because it can improve the effectiveness of conventional treatments. Since the drug’s action in standard formulas is limited by many factors, primarily the low solubility of the drug, its bioavailability is greatly reduced [1]. Furthermore, as patients require administration of high doses of the drug, which is harmful to cells, the therapeutic potential is not sufficiently utilized [2]. Two main strategies are currently used to increase drug hydrophilicity, where it is be encapsulated in amphiphilic copolymers [3,4,5] or conjugated with hydrophilic polymers [6,7,8,9]. They also include the preparation of ionic conjugates. This alternative approach involves attaching drugs by ionic bonding, although these are driven by electrostatic interactions between oppositely charged ions, thus are weaker than the covalent bonds in typical conjugates; however, they appear to be more stable than physical interactions between the encapsulated drug and the polymer matrix. The ionic conjugates can be designed based on poly(ionic liquid)s (PILs) [10,11] as the carriers to prevent too little or too quick drug release. So far, this approach has been used to synthesize linear or graft PILs, which were usually subsequently modified with pharmaceutical anions during ion exchange. Choline-based copolymers with the linear chains containing mefenamic acid [12,13], salicylate [14,15], fusidate, piperacillin, *p*-aminosalicylate, and clavulanate, [16] as well as grafted structures decorated with salicylate [17], clavulanate, *p*-aminosalicylate [18], and fusidate [19], have been explored, showing promising potential in drug delivery. In addition, because of the amphiphilic polymers’ ability to organize into micellar structures, the dual drug systems have been reported for the ionic conjugates of linear PILs to deliver sulfacetamide or salicylate anions and encapsulated quercetin, erythromycin, indomethacin [14], as well as grafted PILs transporting fusidate anions and non-ionic rifampicin [19].

Subsequently, another strategy based on the application of biofunctionalized monomers in the synthesis of ionic drug conjugates has been developed. Initially, ionic liquids (ILs) [20,21], such as phosphonium, imidazolium, lidocaine, pyridinium, ammonium, and choline ones, have been biomodified by incorporation of pharmaceutical counterions through ionic bonding [22], including niflumic, nalidixic, pyrazinoic, and picolinic acids [23], sulfasalazine [24], penicillin-G, amoxicillin [25], ibuprofen [11,26], (acetyl)salicylate [27], and ampicillin [28]. Their biocompatibility has been utilized in medical applications [10,29,30,31], but, in the case of such polymerizable ILs, the polymeric conjugates have been attained [14]. So far, using this strategy, PILs with various topologies bearing salicylate [32], fusidate and/or cloxacillin [33], and *p*-aminosalicylate (PAS) ions [34] have been successfully synthesized.

In this paper, we present an innovative approach to producing ionic graft conjugates bearing PAS anions. While conjugates containing PAS have been previously obtained, such as in designing linear PIL [34] or through post-polymerization modification of graft polymers [18], our method stands out as it involves the direct synthesis of a polymerizable choline methacrylate with a PAS counterion using the *grafting from* technique. Considering the previous results of the amphiphilic nature of the PAS-conjugates, the nanoparticle sizes suitable for drug delivery, and in vitro cytotoxicity tests indicating no significant changes in human bronchial epithelial cells, and adverse effects on the tumor cell line [35], we decided to complete these studies by identifying the characteristics of directly synthesized PAS conjugates with grafted topology. The selected PAS is a well-known antibacterial model drug primarily used to treat the infectious disease *M. tuberculosis*, which affects the lungs [36]. Therefore, in this present work, we discuss a variety of synthetic conditions, which resulted in a series of ionic graft conjugates varied with (i) content of ionic units with PAS anions regulated by initial ratio of choline monomer to comonomer, (ii) side chain length adjusted by initial ratio of monomer to macroinitiator, and (iii) grafting degree provided by the numbers of initiating sites in macroinitiator. The main aim was to understand how to regulate the amount of PAS drug within matrix and the physicochemical properties of the polymer. Furthermore, drug release in vitro tests on chosen carriers were conducted to evaluate the impact of polymer characteristics on the efficiency of drug release and were compared with the other analogous PAS conjugates.

## 2. Materials and Methods

Methyl methacrylate (MMA) was procured from Alfa Aesar (Warsaw, Poland) and dried using molecular sieves (type 4A with a bulk density ranging from 640–670 kg/m^3^, sourced from Chempur in Piekary Śląskie, Poland). The compound [2-(methacryloyloxy)ethyl]trimethylammonium chloride (TMAMA/Cl), available as an 80% aqueous solution from Sigma-Aldrich (Poznań, Poland), was concentrated under a vacuum to produce a solid substance. Copper(I) chloride (CuCl, 98% purity) from Fluka (Steinheim, Germany) underwent purification by being stirred in glacial acetic acid then filtered and washed with both ethanol and diethyl ether, before drying under vacuum. Both methanol (MeOH) and diethyl ether were sourced from Chempur (Piekary Śląskie, Poland). Sodium *p*-aminosalicylate (PAS sodium salt, 98% purity) was used as received without further purification, obtained from Alfa Aesar (Warsaw, Poland). Phosphate-buffered saline (PBS, pH = 7.4), 2,2′-bipyridine (bpy) and tetrahydrofuran (THF) were all purchased from Sigma-Aldrich (Poznań, Poland) and used without further processing.

### 2.1. Synthesis of TMAMA/PAS

**Figure 1:**
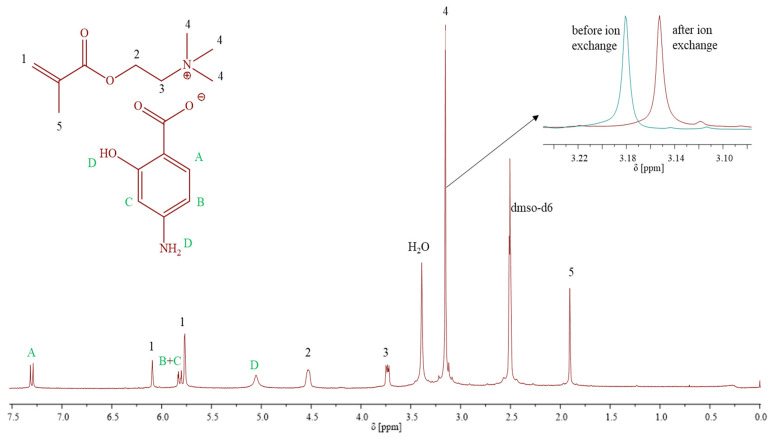
^1^H NMR spectrum of TMAMA/PAS.

Biofunctionalization of TMAMA/Cl to TMAMA/PAS was performed as described previously [34] using the equimolar amounts of PAS sodium salt. The ion exchange process was completed in 4 h, achieving an efficiency of 92%. ^1^H NMR (Figure 1) (DMSO-d6, 300 MHz, δ, ppm): 7.32–7.28 (1H, –CH in aromatic ring), 6.1 and 5.76 (2H, –CH_2_), 5.83–5.77 (2H, –CH in PAS ring), 5.05 (2H, –NH_2_), 4.53 (2H, –CH_2_–O–), 3.74–3.72 (2H, –CH2–N^+^–), 3.15 (9H, –N_+_(CH_3_)_3_), 1.90 (3H, –CH_3_).

### 2.2. Synthesis of Multifunctional Macroinitiators

The copolymers of methyl methacrylate and 2-(2-bromoisobutyryloxy)ethyl methacrylate (P(MMA-*co*-BIEM)) were used as multifunctional macroinitiators (MIs). Their synthesis was detailed in a previous report [18]. Two MIs with various contents of bromoester initiating groups (18% and48 %) were obtained. ^1^H NMR of P(MMA-*co*-BIEM) (DMSO-d6, δ, ppm): 3.66–3.47 (4H, –CH_2_–O–), 3.4–3.26 (3H, –O–CH_3_), 1.94 (6H, –(CH_3_)_2_Br initiating moiety), 2.03–1.65 (2H, –CH_2_– backbone), 1.02–0.56 (3H, –CH_3_ backbone).

### 2.3. Synthesis of Ionic Graft Copolymers Containing PAS Anions (an Example for 8B)

Comonomers TMAMA/PAS (0.151 g, 0.47 mmol) and MMA, (0.05 mL, 0.47 mmol) along with MeOH (0.6 mL), THF (0.45 mL), bpy (1.45 mg, 0.0093 mmol), and MI containing 48% bromoester groups (1.81 mg, including 0.0047 mmol of initiating sites) were added to a Schlenk flask. The mixture was degassed through three freeze-pump-thaw cycles. An initial sample was taken before adding the CuCl catalyst (0.46 mg, 0.0047 mmol) to the mixture. The reaction was allowed to proceed for 19 h, after which it was terminated by exposure to air. The resultant polymer was precipitated twice using a 1:1 *v*/*v* THF-diethyl ether mixture and subsequently dried under a vacuum. ^13^C NMR (DMSO-d_6_, δ, ppm): 172.9 (–C=O, in PAS), 166.7 (–C–C=O), 164.8 (–C=O), 152.3 (H_2_N–C=, in PAS) 131.3 (–C= aromatic, in PAS), 64.3 (–CH_2_-O), 58.8 (–CH_2_–N^+^), 55.6 (O–CH_3_), 53.4 (–CH_2_–, N^+^–(CH_3_)_3_) 40.5 (–C–), 18.4 (–CH_3_). ^1^H NMR (DMSO-d_6_, δ, ppm): 7.32–7.29 (1H, –CH in PAS ring), 5.83–5.77 (2H, –CH in PAS ring), 4.53 (2H, –CH_2_–O–), 3.74–3.71 (2H, –CH_2_–N^+^–), 3.14 (9H, –N^+^–(CH_3_)_3_), 1.90 (3H, –CH_2_), 1.44–0.34 (3H, –CH_3_). FT-IR data: C–N^+^ and C–H (2860–2950 cm^−1^ and 1486 cm^−1^), C=O (1718 cm^−1^), C–N (1633 cm^−1^), C=C aromatic ring (1574 and 1450 cm^−1^), C–H (1385 cm^−1^) C–N (1295 cm^−1^), and C–O ester bonds (1160 cm^−1^).

### 2.4. Drug Release Studies of PAS by Polymer Carrier

Copolymers conjugated with pharmaceutical anions were dissolved in PBS (pH = 7.4) to create solutions with a concentration of 1 mg/mL. Subsequently, 1 mL of this solution was introduced into a dialysis membrane bag with a molecular weight cut-off (MWCO) of 3.5 kDa. This membrane was submerged in a glass vial containing 44 mL of PBS. Throughout the drug release process, conducted at 37 °C, 1 mL samples were periodically collected. These samples were assessed using ultraviolet−visible light spectroscopy (UV–Vis) spectroscopy, noting the absorption maximum peak at λ = 265 nm for PAS. After each measurement, the extracted sample was reintroduced to the glass vial to ensure the PBS medium’s volume remained unchanged. The drug content in the ionic conjugates and the quantity of drug released were determined through the Lambert–Beer law, utilizing the linear calibration curve produced based on the PAS solution in PBS and the relevant absorbance peaks for the anionic drug. The drug quantity in the release medium (*d*) was determined using the subsequent equations:

*d* = *x*/*c* × 100%

*x* = (*y* − *b*)/*a*

where: *a* represents the slope of the calibration curve, *b* stands for the y-intercept of the calibration curve, *x* indicates the concentration value derived from the calibration curve equation, and *y* is the absorbance value at the 265 nm wavelength for PAS.

### 2.5. Characterization

^1^H NMR spectra were recorded using a UNITY/NOVA spectrometer (Varian, Mulgrave, Victoria, Australia), operating at 300 MHz. Samples were measured in deuterated dimethyl sulfoxide (DMSO-d6), using tetramethylsilane (TMS) as the external standard. The molecular weight and dispersity index (represented as M_n_ and Ð, respectively) were determined through size exclusion chromatography (SEC). The SEC measurements utilized an Ultimate 3000 chromatograph (Thermo Fisher Scientific in Waltham, MA, USA), paired with a RefractoMax 521 differential refractometer detector. The polymer samples, diluted in DMF that contained 10 mM LiBr at 50 °C or deionized water, were run through a TSKgel Guard SuperMPHZ-H 6 µm pre-column (4.6 mm × 2 cm) and two TSKgel SuperMultiporeHZ-H 6 µm columns (4.6 mm × 15 cm) at a flow rate of 0.25 mL/min. Calculations were based on poly(ethylene oxide) (PEO) standards ranging from 982 to 969,000 g/mol. The SEC analysis of MIs was conducted using an Ultimate 3000 SEC system, which included an isocratic pump, an autosampler, a degasser, a thermostatic chamber for columns, and utilized a RefractoMax 521 differential refractometer detector. Molecular weight of MIs determined by the RID was calibrated against linear polystyrene standards ranging from 580 to 3,000,000 g/mol. For effective separation, a 5 μm 50 × 7.5 mm pre-column guard and dual PLGel 5 μm MIXED-C and MIXED-D 300 × 7.5 mm columns were employed. These measurements were conducted using HPLC grade THF as the solvent, maintained at 35 °C, with a consistent flow rate of 1 mL/min. Fourier-transform infrared (FT-IR) spectroscopy was performed using a Spectrum Two 1000 FT-IR Infrared spectrometer (Perkin Elmer, Waltham, MA, USA) employing an attenuated total reflection (ATR) method. Throughout the drug release process, samples extracted at specified time intervals were assessed using UV−Vis on an Evolution 300 spectrometer (Thermo Fisher Scientific, Waltham, MA, USA). The quartz cells were used to quantify the drug content and the amount of released pharmaceutical anions.

## 3. Results and Discussion

**Figure 2:**
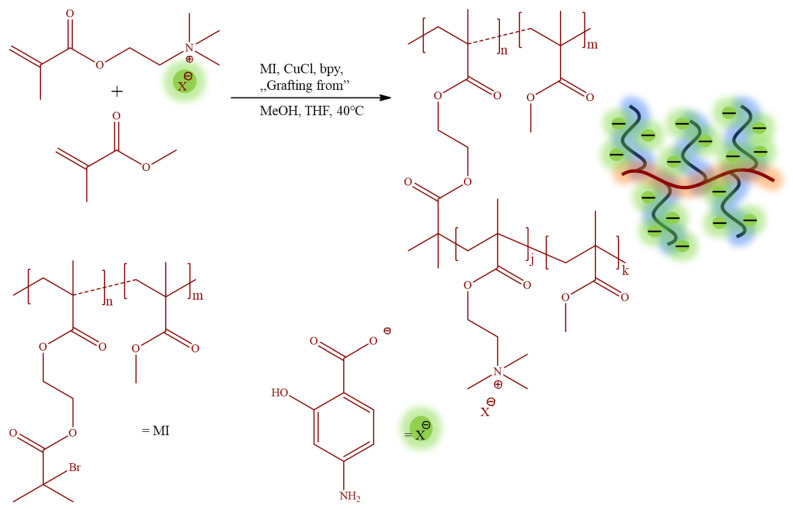
Synthesis of ionic graft copolymers bearing pharmaceutical PAS anions.

**Figure 3:**
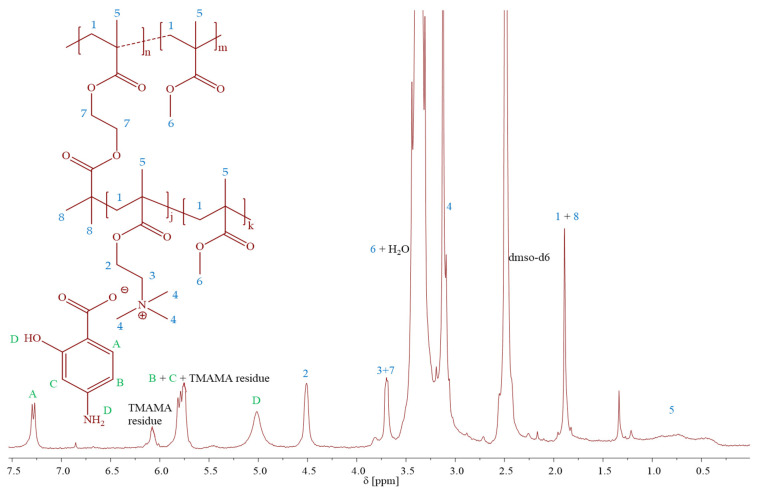
^1^H NMR spectrum of TMAMA/PAS based copolymer.

The monomeric IL [2-(methacryloyloxy)ethyl]trimethylammonium *p*-aminosalicylate (TMAMA/PAS) was employed in the atom radical transfer polymerization (ATRP) using the “grafting from” method to synthesize the well-defined graft copolymers 1A-10B (Table 1). These graft copolymers, as the PAS ionic conjugates providing single drug systems, were designed through the copolymerization of TMAMA/PAS with methyl methacrylate (MMA) in various proportions (25:75, 50:50) in the presence of multifunctional MI, using CuCl/bpy catalytic system at 40 °C in MeOH/THF (Figure 2). The creation of PIL side chains was validated by ^1^H NMR spectra (Figure 3), which displayed signals corresponding to PAS (A-D) and TMAMA part (3 and 4, originating from protons of –CH_2_–N^+^– and –N^+^–(CH_3_)_3_ groups), which were identified previously in the spectra of the related TMAMA/PAS monomer (Figure 1). Concurrently, the signals 1, 5, and 6, associated with the methylene, methyl, and methoxy groups, respectively, showed increased intensity in comparison to the those of MI. The conversions of the monomers were determined using the ^1^H NMR spectrum of the reaction mixture. This involved gauging the integration of signals for the unreacted TMAMA (6.1 ppm) and MMA (6.0 ppm) against the consistent intensity of the pyrene signal (8.19–8.22 ppm), which served as an internal standard.

The polymeric backbone, i.e., P(MMA-*co*-BIEM) was varied in terms of the degree of polymerization (DP_n_ 186 vs. 292) and the number of initiating groups (65 vs. 99 units with the content of 18% vs. 46%, respectively), providing series A and B of the synthesized copolymers. The grafts, i.e., P(TMAMA/PAS-*co*-MMA) presented variations in TMAMA content (F_TMAMA_ = 16–73 mol%), the polymerization degree of side chains (DP_sc_ = 12–89), and the degree of grafting (DG = 18% vs. 46%) defined by the content of initiating groups in the MI.

Because of the solubility of TMAMA/PAS in MeOH and MI in THF, the mixture of these solvents served as the reaction medium. The volume of the polar solvent needed to be higher than the amount of the IL monomer for it to dissolve completely. Simultaneously, the amount of THF had to be well adjusted to avoid precipitation of TMAMA/PAS monomer or the polymer formed during a reaction. Moreover, the MeOH:THF ratio was crucial in minimizing the transesterification of the ionic monomer to MMA, which could occur as a side reaction. In addition, an increased volume of the solvents slowed the reactions. Consequently, durations of most reactions were significantly prolonged to ensure sufficient monomer conversions. Generally, the total solvent volume depended on initial comonomer ratios, M:MI, and number of initiating groups in MI. For reactions with an M:MI ratio of 100:1, ratios of MeOH:TMAMA = 5:1 and MeOH:THF = 5:4 ensured sufficient dissolution of the substrates while effectively inhibiting transesterification for up to 20 h. However, the synthesis of 4B was an exception, where the MeOH:TMAMA ratio was reduced to 4:1 to avoid transesterification. When the M:MI ratio was increased to 200:1 and MI with larger content of initiating groups (48%) was used, the ratio of MeOH:TMAMA = 4:1 was enough to obtain satisfactory solubility of substrates. For the second MI, a greater volume of MeOH at reduced THF volume relative to MeOH became essential for the synthesis of 5A and 6A. These conditions ensured both good solubility and minimal transesterification. In addition, at lower total volume of solvents, the viscosity of these reaction mixtures increased sharply, and cross-linking occurred. The reactions 9B-10B with the decreased ratio of M:MI to 50:1 were performed at a greater overall solvent volume and a distinct excess of MeOH over THF for complete dissolution of reactants. However, these polymerizations had to be stopped after 0.5 or 4 h, respectively, as the reaction mixtures became too viscous because of the formation of densely grafted copolymers using the MI with larger amount of initiating groups.

**Figure 4:**
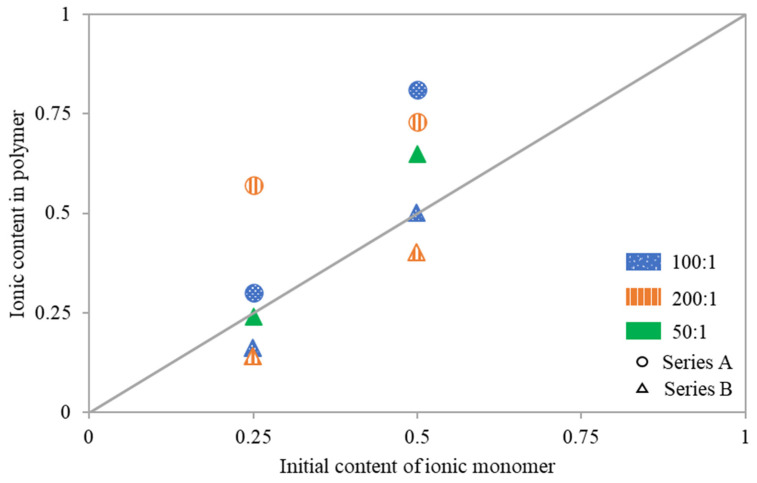
Copolymer composition vs. feed composition of the comonomer mixture.

To analyze reactivity of the ionic monomer copolymerized with MMA the relationship of copolymer composition vs. initial composition of the comonomer mixture were examined (Figure 4). Deviations from the diagonal indicate that the copolymer either had fewer or more ionic fractions than were contained in the initial reaction mixture. Loosely grafted copolymers 5A and 6A, synthesized with an M:MI ratio of 200:1, were significantly richer in TMAMA/PAS units. The same trend was also observed for other reactions performed with initial ratios of TMAMA:MMA = 50:50, that is 2A at M:MI = 100:1 yielding copolymer with lower DG, and 10B at 50/1 and higher DG. Conversely, when using MI containing more active sites at the same conditions as for 5A and 6A, as well as 1A, the ionic contents in resulting polymers 7B and 8B (M:MI = 200:1, TMAMA:MMA = 25:75 and 50:50, respectively), as well as 3B (M:MI = 100:1. TMAMA:MMA = 25:75), were lower than the initial TMAMA/PAS content in the reaction mixture. Meanwhile, for the remaining reactions 1A (M:MI = 100:1, TMAMA:MMA = 25:75), 4B (M:MI = M:MI = 100:1, TMAMA:MMA = 50:50) and 9B (M:MI = 50:1, TMAMA:MMA = 25:75), both monomers converted with similar reactivities, yielding the side chains with copolymer composition comparable to comonomers composition in the initial reaction mixture.

**Figure 5:**
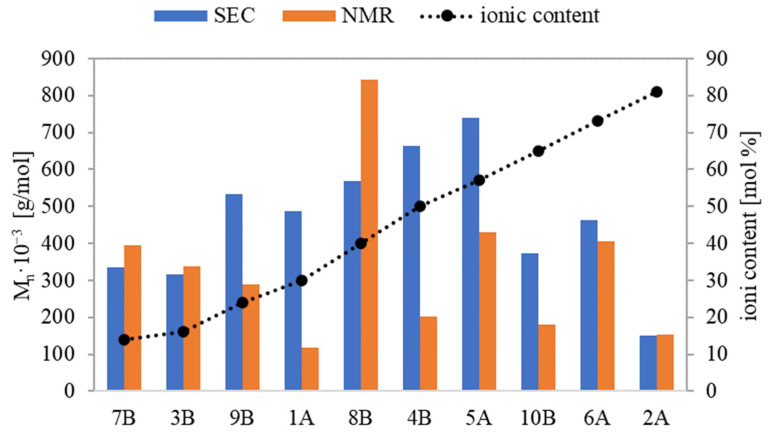
Relationship between M_n, NMR_ and M_n, SEC_ in relation to the ionic fraction content in the graft copolymers carrying PAS.

Based on physicochemical characterization using the SEC method, the polymers’ dispersity indices (Ð) were relatively low, in the range of 1.13-1.46 for series A and 1.3–1.61 for series B. This suggests that the incorporation of the ionic units bearing PAS anions potentially introducing steric hindrance did not diminish the control of ATRP, ensuring dominating homogeneity of graft polymers. There were discrepancies between M_n_ values determined by monomer conversion (M_n,NMR_) and those derived from SEC (M_n,SEC_). The primary explanation is that the latter measurements were taken using a calibration based on linear PEO, which presents a different hydrodynamic volume than the non-linear polymers. For graft copolymers with the lowest and the highest ionic fraction contents (7B and 3B and 6A and 2A, respectively), M_n,NMR_ and M_n,SEC_ were similar (Figure 5). For the remaining copolymers, M_n,NMR_ was generally lower than M_n,SEC_. Possibly due to the aggregation of these ionic conjugates in water, they were running faster through the columns than PEO standards, hence their molecular weights were overestimated. An exception was 8B, containing the longest side chains, where M_n,NMR_ was noticeably higher than M_n,SEC_, probably because of its more rigid, rod-like conformation. In the analogous linear PILs containing PAS, which were characterized with narrower range of index Ð (1.12–1.36), the smallest discrepancy in M_n_ was observed at the highest ionic content [34].

**Figure 6:**
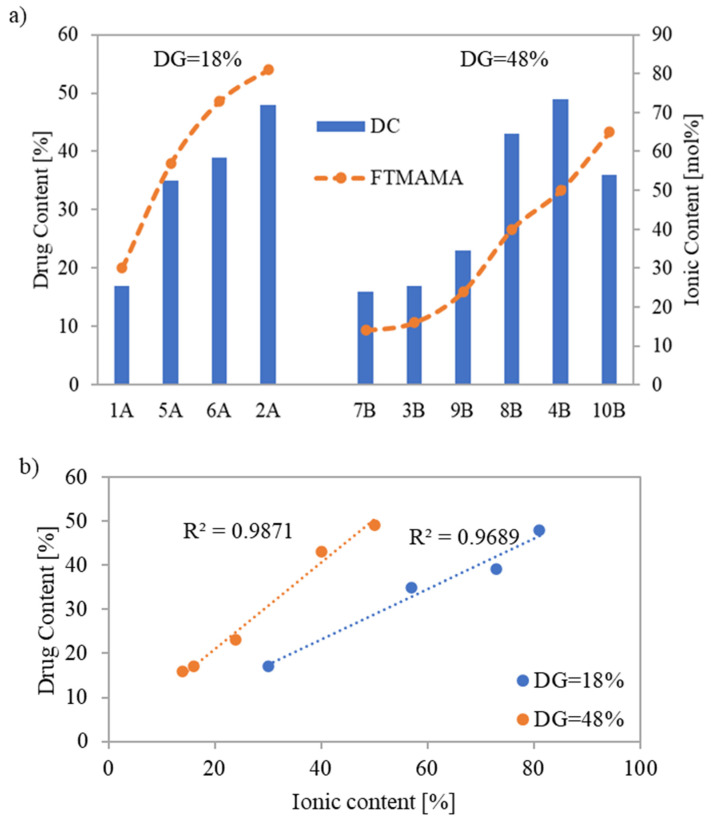
Comparison of F_TMAMA_ and drug content in correlation with DG of copolymers (**a**), and linear relationships between F_TMAMA_ and DC in ionic graft copolymers (**b**).

The drug content (DC) represented by the percentage of PAS incorporated into the copolymer and assessed by UV-Vis spectroscopy ranged between 16% and 49%. The highest DC values, 48% and 49%, were achieved for systems based on conjugates 2A and 4B, respectively (Figure 6a), which were characterized by shorter grafts (DP_sc_ = 21 and 16, respectively). However, copolymer 2A, with a lower number of grafts, demanded a higher F_TMAMA_ (81 mol%) to reach this high DC. Copolymers 6A and 8B which had longer side chains (DP_sc_ = 75 and 89, respectively), also indicated high DC values (39 and 43%, respectively). In this case, for the copolymer 6A with loosely distributed side chains, a higher TMAMA content (73 mol%) was beneficial compared to copolymer 8B with a greater DG and almost double lower F_TMAMA_ to achieve a similar DC. The same correlation was observed when comparing conjugate 1A to 3B or 7B.

The quantity of PAS, introduced during the polymerization of the biomodified TMAMA, increased simultaneously with the ionic unit content in the corresponding linear PILs [34]. This trend was also observed among the graft copolymers with the same grafting density, where a near-linear correlation between ionic content and DC was spotted (Figure 6b). However, there was one exception for conjugate 10B, probably because it had high F_TMAMA_ and the lowest DP_sc_ among all carriers. This tendency was not observed when compared to analogous PAS conjugates formed via polymer modification by exchange with drug anions [18], where the highest DC (64%) was observed for the copolymer with the lowest F_TMAMA_ (18 mol%), indicating the PAS trapping effect within the polymer matrix. Furthermore, the previous copolymers characterized by lower DG = 26% demonstrated DC values (31–64%), which are comparable to the current conjugates with DG = 18%. However, for the previous carriers containing more densely distributed side chains (DG = 46%), drug exchange was restricted to 40%. Regarding systems 4B and 8B (DC = 43% and 49%, respectively), this limit was exceeded, probably due to reduced steric hindrances. In addition, the efficiency of PAS introduction through polymer modification was influenced by the polymer topology, where higher DC values were typically achieved using linear copolymers (DC = 59–82%) [16]. In the case of PAS-conjugates prepared directly from the pharmaceutically functionalized TMAMA, the obtained DC values were comparable, i.e., linear PAS conjugates: DC = 24–42% [34] vs. graft PAS conjugates: DC = 16–49%.

**Figure 7:**
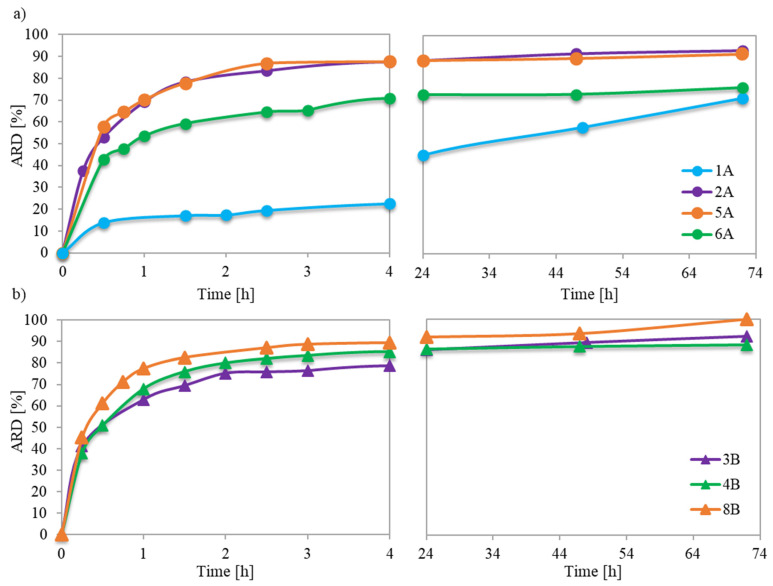
Kinetic release profiles of PAS from ionic copolymers characterized by DG = 18% series A (**a**) and DG = 48% series B (**b**).

Previous in vitro studies investigating the mechanism of PAS release from ionic conjugates have shown that the phosphate anions present in phosphate buffered saline (PBS) can replace the pharmaceutical anions linked to the polymer structure. The capability for such anion exchange largely depended on the specific characteristics of both the polymer and the ionic drug. The studied conjugates with PAS anions of the monomeric units incorporated during the polymerization process revealed their easy availability for anion exchange, which resulted in amount of released drug (ARD) values ranging from 71% to 100% (Table 2). The immediate burst release persisted for about 1.5 to 2.5 h, providing a logarithmic growth of ARD (Figure 7a,b) and exchange of a major of the PAS, after which an extra 4–15% of drug was released. However, polymer 1A exhibited a unique behavior, yielding an initial exchange of 17% of PAS, and continuation for up to 72 h resulted in the release of an additional 54% of the ionic drug. However, the concentration of released drug (CD) was the lowest among all carriers. The extended-release time in this case can be attributed to the weaker ionic interactions between the PAS anions and the matrix of the loosely grafted copolymer with lower drug content.

**Figure 8:**
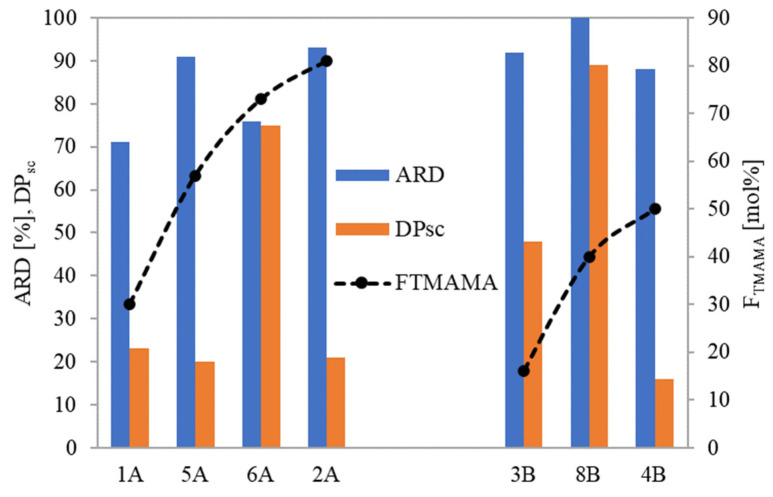
Dependence of amount of released drug on polymer characteristics.

For loosely grafted brushes, the ARD typically increased with the ionic content (and consequently DC), as illustrated in Figure 8. However, conjugate 6A deviated from this correlation, likely due to the PAS anions being more hidden within its long side chains. In the context of brushes with densely packed side chains, they exhibited high ARD regardless of F_TMAMA_ or DP_sc_. This is plausible because at higher graft density, there were stronger repulsive interactions between the PAS ions. This made them more available for an ion exchange. Notably, the maximum ARD from 8B was attained as its longer side chains further intensified these repulsive interactions. 

The smaller ARD from the previously reported graft conjugates, which have been prepared via anion exchange modification of polymer, ranged between 33% and 45% [18]. In those cases, burst and effective release of PAS was also evident up to 1.5 h, with the release persisting until 4 h after which a plateau was reached. The initial burst release indicated that the drug was primarily localized in the outer regions of the polymer, as it was detected for the currently studied graft copolymers, while the remaining drug anions were embedded deep within the polymer structure. Probably during anion exchange on the polymer, this part of the drug anions was internally trapped, as was observed for encapsulation, what made it challenging for them to diffuse through the entangled polymer matrix. In addition, graft topology slowed down drug exchange in comparison to the corresponding linear P(TMAMA/PAS–*co*–MMA)s, from which complete PAS release occurred within 1–4 h [34].

## 4. Conclusions

The choline-based monomeric IL, biofunctionalized with pharmaceutically active PAS anions, was applied to synthesize the well-defined ionic graft copolymers as the ionic conjugates of drug. They had varied ionic unit contents (14–81 mol%) and the anionic drug contents (16–49%) in the side chains. In the case of grafting from a macroinitiator containing less active sites, the resultant polymers featured more ionic units compared to the TMAMA content in the initial monomer mixture, whereas for densely grafted brushes, the ionic content in the polymer was usually similar or lower than the initial TMAMA content. Copolymers comprising loosely spaced side chains required a significantly higher TMAMA content to achieve drug content comparable to that of densely grafted brushes with lower ionic content, regardless of the side chain length. The in vitro drug release kinetics exhibited a burst release phase lasting up to 2.5 h, during which the majority of the drug was typically released. The released amounts of PAS ranged from 71% to 100% (2.8–10.2 μg/mL). Regarding densely grafted brushes, neither TMAMA content nor length of side chains impacted the drug delivery efficiency, but for the loosely grafted copolymers with shorter side chains, amount of released drug increased with the ionic content. It is worth noting that these systems released significantly more drug compared to analogous PAS conjugates with similar drug content prepared via pharmaceutical modification of polymer. In essence, the direct synthesis of ionic drug conjugates from the pharmaceutically modified TMAMA monomer appeared to be an effective strategy to procure promising materials for PAS delivery applications. In the future, we plan to exploit the amphiphilic character of these copolymers and their ability to form nanoparticles to encapsulate additional drug to develop PAS systems for a combination therapy.

## Figures and Tables

**Table 1:** Characteristics of graft copolymers carrying PAS.

No.	Time [h]	M:MI	[TMAMA: MMA]_0_	MEOH: TMAMA v:wt	MeOH: THF: *v*/*v*	^1^H NMR			SEC		UV-Vis
						DP_sc_	F_TMAMA_ [mol%]	M_n,NMR_ 10^−3^ [g/mol]	M_n,SEC_ 10^−3^ [g/mol]	Ð	DC [%]
1A	20	100:1	25:75	5:1	5:4	23	30	117.1	486.6	1.28	17
2A	20		50:50	5:1	5:4	21	81	153.4	149.2	1.13	48
3B	20		25:75	5:1	5:4	48	16	338.5	317.1	1.49	17
4B	18		50:50	4:1	4:3	16	50	201.5	663.4	1.3	49
5A	4	200:1	25:75	8:1	8:5	35	57	431.3	738.7	1.36	35
6A	4		50:50	6:1	6:4	75	73	405.0	463.3	1.46	39
7B	21		25:75	4:1	4:3	56	14	393.7	334.5	1.34	16
8B	19		50:50	4:1	4:3	89	40	841.9	567.7	1.4	43
9B	0.5	50:1	25:75	7:1	7:4	34	24	289.2	532.6	1.36	23
10B	4		50:50	6.5:1	6.5:4	12	65	179.5	372.9	1.61	36

Where: M:MI = [TMAMA/PAS+MMA]_0_:[MI]_0_; [MI]_0_:[CuCl]_0_:[bpy]_0_ = 1:1:2, MI for series A contains 65 initiating groups (18%), and for series B: 99 initiating groups (48%), DP_sc_—the polymerization degree of the side chains, F_TMAMA_—content of TMAMA in the side chains, SEC: determined in water, PEO calibration.

**Table 2:** Data for PAS released from ionic graft conjugates.

No.	CD [μg/mL]	ARD [%]
1A	2.8	71
2A	10.2	93
3B	3.5	92
4B	9.9	88
5A	8.8	91
6A	8.4	76
8B	9.8	100

## Data Availability

The data presented in this study are available in this article.
